# Protective effects of tumor necrosis factor-α blockade by adalimumab on articular cartilage and subchondral bone in a rat model of osteoarthritis

**DOI:** 10.1590/1414-431X20154407

**Published:** 2015-07-31

**Authors:** C.H. Ma, Q. Lv, Y.X. Yu, Y. Zhang, D. Kong, K.R. Niu, C.Q. Yi

**Affiliations:** 1 Department of Orthopedic Surgery, Shanghai First People's Hospital, Shanghai Jiao Tong University, Shanghai, China; 2 Department of Radiology, Tong Ji Hospital, Tong Ji University, Shanghai, China

**Keywords:** Anti-tumor necrosis factor-α antibody (ATNF), Osteoarthrosis, Matrix metalloproteinase (MMP)-13, Articular cartilage, Subchondral bone

## Abstract

We aimed to investigate the effects of an anti-tumor necrosis factor-α antibody (ATNF) on cartilage and subchondral bone in a rat model of osteoarthritis. Twenty-four rats were randomly divided into three groups: sham-operated group (n=8); anterior cruciate ligament transection (ACLT)+normal saline (NS) group (n=8); and ACLT+ATNF group (n=8). The rats in the ACLT+ATNF group received subcutaneous injections of ATNF (20 μg/kg) for 12 weeks, while those in the ACLT+NS group received NS at the same dose for 12 weeks. All rats were euthanized at 12 weeks after surgery and specimens from the affected knees were harvested. Hematoxylin and eosin staining, Masson's trichrome staining, and Mankin score assessment were carried out to evaluate the cartilage status and cartilage matrix degradation. Matrix metalloproteinase (MMP)-13 immunohistochemistry was performed to assess the cartilage molecular metabolism. Bone histomorphometry was used to observe the subchondral trabecular microstructure. Compared with the rats in the ACLT+NS group, histological and Mankin score analyses showed that ATNF treatment reduced the severity of the cartilage lesions and led to a lower Mankin score. Immunohistochemical and histomorphometric analyses revealed that ATNF treatment reduced the ACLT-induced destruction of the subchondral trabecular microstructure, and decreased MMP-13 expression. ATNF treatment may delay degradation of the extracellular matrix via a decrease in MMP-13 expression. ATNF treatment probably protects articular cartilage by improving the structure of the subchondral bone and reducing the degradation of the cartilage matrix.

## Introduction

Osteoarthritis (OA) is a highly prevalent aging-associated degenerative joint disorder in middle-aged and older people around the world (1). Clinically, the disease is characterized by progressive degeneration of articular cartilage with inflammation in the synovium, subchondral bone sclerosis, and marginal osteophyte formation (2). Although many factors contribute to the onset of OA, including genetic, metabolic, biochemical, and biomechanical factors (3,4), the exact pathogenesis of OA remains unknown (5). The main approach in current OA therapies is drug treatment to relieve pain and improve joint function. However, there are no therapeutic strategies that can address the underlying causes to halt OA progression.

Chondrocytes and extracellular matrix (ECM) are the pivotal structural components of cartilage (6). OA is considered to start as a result of damage to the joint tissue by physical forces such as single or repeated microtrauma (7). Chondrocytes respond to such physical injury by stopping the production of anabolic factors and by releasing more catabolic enzymes such as matrix metalloproteinases (MMPs). These responses result in further damage to the cartilage (8), and consequently lead to the release of matrix components, which elicit inflammatory mechanisms. Moreover, breakdown of the ECM eventually presents as articular cartilage degeneration (6). Studies have revealed that the pathophysiology of OA involves proinflammatory cytokines, such as interleukin-1β, tumor necrosis factor (TNF)-α, and interleukin-6 (9). The role of TNF-α in the pathogenesis of OA has drawn increasing attention in recent years because of its predominance in the pathogenesis of other arthritic diseases (10-12). By retarding joint damage, approaches that target TNF-α blockade may provide effective therapies for OA.

Adalimumab (D2E7; Abbott Laboratories, USA) is the first fully human (100% human peptide sequences) monoclonal antibody that blocks TNF-α. Regarding rheumatoid arthritis (RA), adalimumab is currently being evaluated in clinical trials because it can slow progressive joint destruction (13,14). Few studies have explored the effects of adalimumab therapy on OA in clinical settings. Verbruggen et al. (15) showed that adalimumab significantly delayed the progression of joint damage in patients with erosive hand OA. However, the mechanism underlying the effects of adalimumab on OA is largely unknown. Therefore, further studies are needed to clarify the exact effects of adalimumab on OA.

The anterior cruciate ligament transection (ACLT) model is one of the most widely used models of OA, and mimics early OA in humans very well (16-18). In the present study, we examined the potential effects of an anti-TNF-α antibody (ATNF) by observing the changes in the chondrocytes, ECM, and subchondral trabecular bone in the rat ACLT model of OA. We aimed to explore the potential mechanism underlying the effects of ATNF on OA. We hypothesized that ATNF could affect TNF-α-induced MMP-13 expression and improve the subchondral bone microstructure to inhibit cartilage degeneration and alter the subchondral bone quality. Our study can provide a theoretical basis for the potential effects of ATNF on OA in clinical trials.

## Material and Methods

### Animal handling and ACLT surgery

All procedures were approved by the Animal Care and Ethics Committee of Shanghai First People's Hospital, Shanghai Jiao Tong University. A total of 24 healthy female Sprague-Dawley rats (aged 11 weeks, weighing 272.5±38.5 g; Charles River Corporation, China; Batch No. SCXK Beijing, 2007-0001) were used in the following experiments. The rats were randomly separated into three groups of eight rats as follows: sham-operated (SP), ACLT+normal saline (NS) (ACLT+NS); and ACLT+ATNF.

The rats in the ACLT+ATNF and ACLT+NS groups underwent ACLT surgery, while the rats in the SP group underwent knee joint exposure only, followed by suturing. The ACLT model was created as previously described (19). Briefly, each rat in the ACLT+ATNF and ACLT+NS groups was anesthetized intraperitoneally with 10% chloral hydrate (30 mg/kg) prior to surgery. Their right legs were prepared and draped in a standard sterile manner, before an approximately 2-cm midline incision was made over the knee. The patella was dislocated laterally and the knee placed in full flexion. The anterior cruciate ligament was transected with micro-scissors, and complete transection was confirmed by the anterior drawer test. For surgery in the SP group, the knee joint space was exposed, but the anterior cruciate ligament was not transected. Following surgery, the capsule and skin were both sutured, and the skin was disinfected with iodine.

At 3 days after surgery, the rats were given an antibiotic (50,000 U penicillin/rat) every day with appropriate postoperative care and allowed to exercise freely. After 1 week, the rats in the ACLT+ATNF group were given ATNF (adalimumab) treatment every 2 days by subcutaneous injection at a dosage of 20 μg/kg. The rats in the ACLT+NS group were injected with NS using the same volume as the ACLT+ATNF group. The rats were checked daily (activity, body weight, food consumption, rectal temperature, wound healing). All animals were treated for 12 weeks and then euthanized.

### Specimen collection

The rats were euthanized by cervical dislocation and their right knee joint spaces were opened. The gross appearance of the distal femur was observed and recorded with a digital camera (Model 550D; Canon, Japan). Specimens of the femurs were then fixed in 4% paraformaldehyde for 72 h, treated with 15% disodium ethylenediaminetetraacetic acid, and embedded in paraffin. The embedded tissues were sectioned at 4-μm thickness and processed for conventional staining, specific staining, and immunohistochemistry.

In addition, specimens of the proximal end of the tibias were fixed in 70% ethanol for 72 h. The tissues were then dehydrated, embedded in methyl methacrylate, cut into 5-μm thick sections, and subjected to von Kossa staining. Using light microscopy, two color digital images were recorded to analyze the articular cartilage lesions in sections from different regions. For analysis of morphological changes to the cartilage, the Mankin score system was properly adjusted and applied.

### Bone histomorphometry

Undecalcified samples were subjected to von Kossa staining (20). Histomorphometric measurements were determined with a DMLB2 fluorescence/light microscope (Leica, Germany) and a DC300 figure shoot system (Leica, Germany). A Leica Qwin image analysis system with automated analysis was used for evaluation of standard morphological parameters, including bone volume fraction (BV/TV), trabecular bone thickness (Tb.Th), trabecular bone number (Tb.N), and trabecular bone separation (Tb.Sp).

### Hematoxylin and eosin staining

Sections were deparaffinized by sequential immersion in xylene, rehydrated in solutions of absolute alcohol (95%, 80%, and 70% alcohol), and briefly washed in distilled water. The sections were stained in Harris hematoxylin solution for 5 min, differentiated in 0.5% acid alcohol for 1 min, and stained blue in ammonia water. Counterstaining was performed in eosin solution for 1 min. The stained sections were dehydrated through a graded series of alcohol, cleared with xylene, and mounted with neutral balsam. The morphology of the tissues was observed using an Olympus BX40 light microscope (Olympus, Japan). The sections were scored using slightly modified Mankin criteria (21) (Table 1).

**Table:**
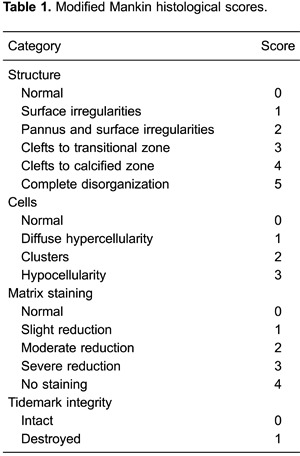

### Masson's trichrome staining

Masson's trichrome staining was performed as described previously (22). Briefly, the sections were deparaffinized, hydrated to distilled water, oxidized in 1% potassium permanganate solution, and rinsed with tap water. After bleaching with oxalic acid for 1 min, washing in distilled water, and staining in Celestine blue for 5 min, the sections were stained in Mayer's hematoxylin solution for 3–5 min and rinsed in running tap water for 5–10 min. The sections were then stained in Ponceau-picric acid saturated solution for 5 min, rinsed in 1% acetic acid-water, differentiated in 1% phosphomolybdic acid for 5 min, rinsed in distilled water, stained in 1% light green or toluidine blue for 30 s, rinsed in 1% acetic acid-water, differentiated in 95% alcohol, hydrated in absolute alcohol, cleared with xylene, and mounted with neutral balsam.

### Immunohistochemical staining of MMP-13

To clarify the molecular mechanism underlying cartilage degeneration, MMP-13 expression was detected by immunohistochemical staining. Paraffin sections were baked for 20 min, routinely deparaffinized, hydrated to distilled water, and washed three times in phosphate-buffered saline (PBS) for 3 min each. The sections were then treated with complex enzyme digestion for antigen retrieval for 15 min at room temperature, incubated with 3% H_2_O_2_solution for 10 min, and washed three times with PBS for 3 min each. Next, the sections were overlaid with 30 µL of anti-mouse MMP-13 polyclonal antibody (Boster Corporation, China) for 10 min at room temperature, washed three times with PBS for 3 min each, incubated with 30 µL of biotinylated goat anti-mouse secondary antibody (DAKO, Denmark) for 40 min, washed three times with PBS for 3 min each, stained with 50 µL of 3,3′-diaminobenzidine solution with appropriate termination, and rinsed in tap water for 3 h. Counterstaining was carried out with hematoxylin for 5 min. The sections were then dehydrated through a graded series of alcohol for 10 s each, cleared with xylene, and mounted with neutral balsam. Images magnified 400-fold were obtained, and the integrated absorbance was determined by Image Pro Plus (IPP) software (Media Cybernetics, USA) to quantify the protein expression.

### Statistical analysis

Results were analyzed using the Shapiro-Wilk normality test (23) and Bartlett homogeneous variance test (24). Student's *t*-test was used to assess the statistical differences among groups and Duncan's multiple-range test was used for values of two individuals. Data were reported as means±SD. Values of P<0.05 were considered to indicate statistical significance.

## Results

### Gross macroscopic assessment

**Figure 1:**
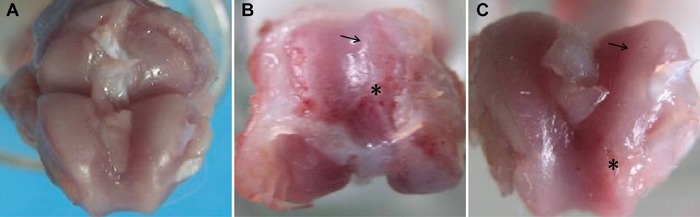
Representative images of femoral condyles at 12 weeks after surgery. *A*, The sham-operated (SP) group shows a normal appearance. *B*, In the anterior cruciate ligament transection (ACLT)+normal saline (NS) group with NS treatment after ACLT, the joints developed surface damage (arrow) and severe osteophytes (asterisk). *C*, The ACLT+anti-tumor necrosis factor-α antibody (ATNF) group shows a significant reduction in surface damage (arrow) and smaller osteophytes (asterisk).

All animals recovered quickly after surgery, and there were no obvious differences in body weight of the rats among the SP, ACLT+ATNF, and ACLT+NS groups (data not shown). The gross appearances were recorded, as shown in Figure 1.

In the SP group, the knees of the rats exhibited translucent smooth articular surface, resembling healthy cartilage, and no osteophyte formation in the femoral condyles or tibial plateaus. The synovial fluid of the knee joints was clear (Figure 1A). In the ACLT+NS group, the knee joints swelled, the cartilage displayed rougher surface, local ulceration and erosion, the synovial fluid was yellow and opaque, the femoral condyles appeared to be hypertrophic, and osteophytes could be found, comprising the typical appearance of OA (Figure 1B). In the ACLT+ATNF group, the majority of the cartilage surface showed slightly rough appearance without osteophyte formation, the synovial fluid was light yellow, and cartilage degradation was decreased compared with the ACLT+NS group (Figure 1C). Overall, the joints in the ACLT+ATNF group showed moderate cartilage degeneration compared to the ACLT+NS group.

### Histomorphometric analysis of subchondral bone

Von Kossa staining was performed for bone histomorphometric analysis. Compared with the ACLT+NS group, BV/TV and Tb.N of the rats in the ACLT+ATNF group were significantly increased while Tb.Sp was markedly decreased (P<0.05). However, no significant differences in these parameters were observed between the SP group and the ACLT+ATNF group (Table 2).

**Table:**
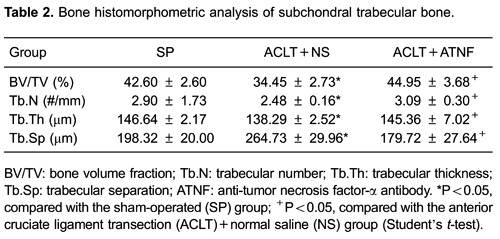

### Cartilage cell morphology

**Figure 2:**
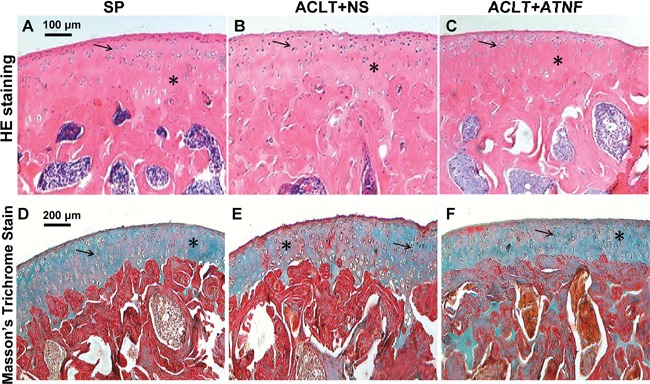
Histological comparison of articular cartilage findings at the distal femur by hematoxylin and eosin (HE) staining (magnification 200×) and cartilage matrix morphology findings by Masson's trichrome staining (magnification 100×). *A*, *D*, sham-operated (SP) group; *B*, *E*, anterior cruciate ligament transection (ACLT)+normal saline (NS) group; *C*, *F*, ACLT+anti-tumor necrosis factor-α antibody (ATNF) group. In the ACLT+NS group, the cells had an irregular arrangement. In the ACLT+ATNF group, the cells re-established an ordered pattern and the cartilage matrix was slightly and unevenly stained. Arrows indicate the cells. Asterisks represent the cartilage matrix.

**Figure 3:**
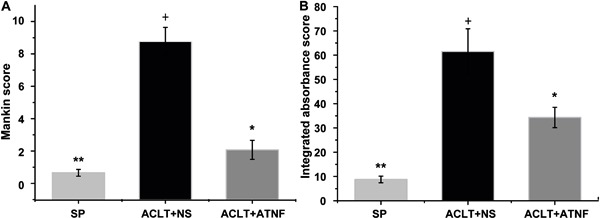
*A,* Mankin scores for grading of cartilage lesions. *B,* integrated absorbance values reflecting matrix metalloproteinase (MMP)-13 expression. ^+^P<0.05, anterior cruciate ligament transection (ACLT)+normal saline (NS) group compared to the sham-operated (SP) group; *P<0.05, ACLT+anti-tumor necrosis factor-α antibody (ATNF) group compared to the ACLT+NS group; **P<0.05, SP group compared to the ACLT+ATNF group (Student's *t*-test).

Hematoxylin and eosin (HE)-stained sections were used to score samples for features of cartilage pathology, including changes in cellularity and structural abnormalities (Figure 2). In the SP group, the cartilage of the rats appeared as a thick neatly arranged layer, and the staining was normal. The boundary between the calcified cartilage and subchondral bone was intact. In the ACLT+NS group, cartilage lesions were severe. The cartilage layer was thinner, with cell loss, cell cloning, and multicellular chondrocyte clusters, and overall the cells appeared in a less ordered structure. The subchondral bone invaded the calcified cartilage. However, in the ACLT+ATNF group, there were no significant lesions, slight erosion was observed in the cartilage surface, and the cells re-established an ordered pattern, in which the cells were increased in the superficial zone and flattened in the transitional zone. Taken together, these changes resulted in Mankin grades reflected by the analyses shown in Figure 3A.

The Mankin score in the ACLT+NS group was dramatically higher than that in the SP group, and significantly higher than that in the ACLT+ATNF group.

### Cartilage matrix morphology

The cartilage ECM alterations were evaluated by Masson's trichrome staining (Figure 2). Masson trichrome commonly stains the cartilage matrix blue, the nuclei dark blue, and the zone of calcifying cartilage red. We found that the SP group had a regular cell arrangement and dark staining. In the ACLT+NS group, red staining was found, the matrix was strongly but unevenly stained, and the cells had an irregular arrangement. In the ACLT+ATNF group, the cartilage matrix was slightly and unevenly stained, the cells were in an ordered arrangement, and red staining was reduced in the articular cartilage compared with that in the ACLT+NS group.

### Immunohistochemical analysis

**Figure 4:**
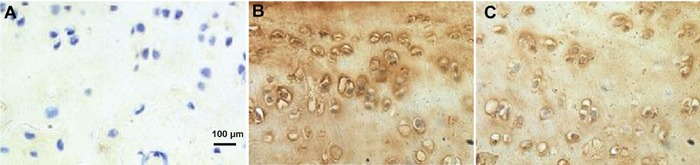
Immunohistochemical analysis of anti-tumor necrosis factor-α antibody (ATNF) effects on matrix metalloproteinase (MMP)-13 in cartilage lesions (original magnification 400×). *A*, Staining for MMP-13 is less detectable in the sham-operated (SP) group. *B*, In the anterior cruciate ligament transection (ACLT)+normal saline (NS) group, MMP-13 expression is found throughout the articular cartilage. *C*, In the ACLT+ATNF group, MMP-13 is mainly detected in chondrocytes at and close to the articular surfaces.

Immunohistochemical staining for MMP-13 expression is shown in Figure 4. Staining for MMP-13 was less detectable in the SP group. In the ACLT+ATNF group, MMP-13 was mainly detected in chondrocytes at and close to the articular surfaces (Figure 4C). In the ACLT+NS group as a control, MMP-13 expression was found throughout the articular cartilage. In the ACLT+ATNF group, ATNF treatment reduced the expression of MMP-13 in cartilage and the integrated absorbance values of the positive cells in the cartilage of rats in the SP group were markedly lower than those in the ACLT+NS group. The integrated absorbance values of positive cells in the cartilage of the ACLT+ATNF group were reduced compared with those in the ACLT+NS group (Figure 3B).

## Discussion

OA is a common joint disease in the elderly and impedes their daily life. Degenerative alterations to the cartilage and subchondral bone play key roles in OA development (25). Our study demonstrated that ATNF treatment can inhibit cartilage degradation by decreasing MMP-13 expression related to the modulation of cartilage metabolism in a rat model of OA. In addition, ATNF treatment ameliorated the subchondral trabecular bone alterations in the knee joints induced by ACLT injury compared with those in the ACLT+NS group.

Numerous studies support that the entire synovial joint is involved in OA, with alterations occurring in the articular cartilage, subchondral bone, capsule, ligaments, periarticular muscles, and synovial membrane (26,27). However, articular cartilage is the major target of tissue injury with ulceration, fissures, and full-thickness loss from the joint surface (27). OA degeneration is also characterized by extensive joint remodeling, which is often associated with the formation of new bone (osteophytes) at the joint margins, increased subchondral plate thickness, and sclerosis (28). The rat ACLT model can only mimic some features of human OA, because human OA is more complex and has different phases. However, articular cartilage degeneration is a hallmark of OA. Furthermore, recent studies have characterized the cartilage degradation in the rat ACLT model (29). In line with the previous studies, our study confirmed the effects of ATNF treatment on OA, based on gross observations revealing that the majority of the cartilage surface had a smooth surface in the ACLT+ATNF group.

Knee instability following ACLT often induces OA accompanied by degradation of the articular cartilage matrix (29). Besides, Jean et al. (30) demonstrated that breakdown of the cartilage matrix can lead to fissures, fibrillation, gross ulceration, and even full-thickness loss at the joint surface. Aigner et al. (31) described that HE staining of OA cartilage shows surface fibrillation, typical chondrocyte clustering, and typical chondrocytes. Moreover, another study showed that TNF-α suppresses matrix synthesis by chondrocytes, which is essential for adequate matrix function and balance in RA and OA (32). In our study, we used HE staining and a modified Mankin score to evaluate the cartilage degeneration. Increases in the Mankin score indicated that degenerative changes had occurred (Figures 2 and 3). We also showed that both the macroscopic and Mankin scores were significantly lower in the ACLT+ATNF group than in the ACLT+NS group. Moreover, reduction in the severity of the structural changes and less Masson trichrome staining were observed in the ACLT+ATNF group. In addition, the present findings confirm and extend previous observations on the calcified zone of cartilage in experimental rat OA. Thus, we suggest that ATNF treatment may directly contribute to chondrocyte proliferation and ECM integrity.

Subchondral bone and articular cartilage act as a functional unit in joints (33). Subchondral bone has been shown to be an interesting target in OA treatment (34). Through microscopic observations, Radin et al. (35) showed that subchondral trabecular microdamage could aggravate cartilage degradation in OA. Muraoka et al. (36) reported that before the onset of cartilage degeneration in OA, the subchondral bone was fragile, and had low BV/TV and Tb.Th, and high Tb.Sp. In our study, the histomorphometric analysis of subchondral bone showed that ATNF treatment markedly increased the BV/TV, Tb.Th, and Tb.N (Table 2), indicating that the subchondral bone microstructure was improved. Therefore, our results suggest that ATNF treatment might alter the subchondral bone quality by improving the subchondral trabecular microstructure in this rat model of OA.

MMPs are the major mediators of cartilage destruction and can break down the components of the ECM (37). Goldring et al. (38) demonstrated that MMP-13, which is the major type II collagen-degrading collagenase in cartilage ECM and can be induced by interleukin-1β and TNF-α, contributed not only to irreversible joint damage in OA, but also to the initiation/onset phase. In addition, another study revealed that TNF-α regulates MMP expression through signal transduction pathways, such as the nuclear factor-κB pathway (32). Besides, Kanbe et al. (39) demonstrated that adalimumab treatment for RA could decrease MMP-3 expression in the synovium. In the present study, immunohistochemical analyses demonstrated that ATNF treatment reduced the expression of MMP-13, and that both the numbers and integrated absorbance values of positive cells were lower in the ACLT+ATNF group than those in the ACLT+NS group. Taken together, the present findings suggest a possible mechanism for adalimumab in counteracting OA that involves prevention of cartilage degeneration by inhibiting MMP-13 expression.

In summary, our findings strongly suggest that ATNF counteracts the histomorphological cartilage degeneration and subchondral bone loss associated with OA by decreasing the MMP-13 expression and improving the subchondral bone microstructure in a rat model of OA. The protective effects of ATNF treatment raise the possibility that this form of treatment may have therapeutic benefits for humans with knee OA, in agreement with the few previous studies in clinical settings. However, the present study cannot explain the concrete mechanism underlying the ATNF-induced decrease in MMP-13 expression, and further efforts are needed to clarify this mechanism in future studies. Taken together, the present study suggests that adalimumab is a good candidate for limiting the pathological progress in OA.
